# Behavioral and EEG Evidence for Auditory Memory Suppression

**DOI:** 10.3389/fnhum.2016.00133

**Published:** 2016-03-30

**Authors:** Maya E. Cano, Robert T. Knight

**Affiliations:** ^1^Helen Wills Neuroscience Institute, University of California, BerkeleyBerkeley, CA, USA; ^2^Department of Psychology, University of California, BerkeleyBerkeley, CA, USA

**Keywords:** think/no-think, EEG, forgetting, auditory memory, directed forgetting, memory suppression

## Abstract

The neural basis of motivated forgetting using the Think/No-Think (TNT) paradigm is receiving increased attention with a particular focus on the mechanisms that enable memory suppression. However, most TNT studies have been limited to the visual domain. To assess whether and to what extent direct memory suppression extends across sensory modalities, we examined behavioral and electroencephalographic (EEG) effects of auditory TNT in healthy young adults by adapting the TNT paradigm to the auditory modality. Behaviorally, suppression of memory strength was indexed by prolonged response time (RTs) during the retrieval of subsequently remembered No-Think words. We examined task-related EEG activity of both attempted memory retrieval and inhibition of a previously learned target word during the presentation of its paired associate. Event-related EEG responses revealed two main findings: (1) a centralized Think > No-Think positivity during auditory word presentation (from approximately 0–500 ms); and (2) a sustained Think positivity over parietal electrodes beginning at approximately 600 ms reflecting the memory retrieval effect which was significantly reduced for No-Think words. In addition, word-locked theta (4–8 Hz) power was initially greater for No-Think compared to Think during auditory word presentation over fronto-central electrodes. This was followed by a posterior theta increase indexing successful memory retrieval in the Think condition. The observed event-related potential pattern and theta power analysis are similar to that reported in visual TNT studies and support a modality non-specific mechanism for memory inhibition. The EEG data also provide evidence supporting differing roles and time courses of frontal and parietal regions in the flexible control of auditory memory.

## Introduction

Memory is an essential part of our cognitive lives, but it is becoming increasingly evident that forgetting also plays an important role in successful functioning. Many memories decay over time (Ebbinghaus, 1913), but what is not yet well understood is how this process occurs. Traditionally, the act of forgetting has been treated as a passive process, characterized simply as a failure to remember or refresh events (i.e., failure to encode or retain information), but recent work suggests that prefrontal dependent processes are engaged in active forgetting. Although forgetting often carries a bad connotation, the act of forgetting can have clear adaptive benefits. For instance, an inability to forget would result in an overwhelming amount of stored irrelevant information, which would interfere with encoding and retrieval of relevant information. Furthermore, some memories may be harmful to an individual, as seen in post-traumatic stress disorder (PTSD). Forgetting or even reducing the strength of these memories would be beneficial. Given the utility of this forgetting process, a thorough understanding of its mechanisms is warranted.

The process of forgetting has been addressed in various ways. For example, retrieval-induced forgetting, in which the retrieval of a particular item inhibits the memory for or the ability to retrieve related items, has been proposed as a mechanism of forgetting (Anderson et al., 1994). Additionally, directed forgetting paradigms using both the list (MacLeod, 1975) and item methods (Elmes et al., 1970), have been utilized to investigate how the instruction to forget certain stimuli affects later memory recall. However, processes that are unrelated to memory inhibition could explain findings of successful forgetting in these types of studies. For instance, retrieval-induced forgetting is potentially due to automatic interference at the time of recall, while memory failures in directed forgetting tasks could result from unsuccessful shallow encoding. Although these studies have shown that forgetting can be manipulated, they do not offer a sufficient model for the intentional inhibition of memory.

Anderson and Green (2001) introduced the concept of forgetting as an explicit and controllable active process with the development of the Think/No-Think (TNT) paradigm—a modified Go/No-Go task that probes more specifically how already formed memories can be selectively enhanced or suppressed. In this task, subjects first learn word pairs and then are instructed to either retrieve or suppress the second item in a pair when presented with the first item of that pair. Critically, a subset of the learned pairs is not seen again until a surprise subsequent memory retrieval test, and serves as a baseline for passive forgetting. The “Think” items (i.e., those that are submitted to practiced retrieval during the task) were found to be better recalled as a function of trial repetition, such that the more times a word was retrieved, the more likely it was to be remembered in a later memory test. The surprising and important finding was that memory inhibition practice also aided in forgetting. That is, the suppressed or “No-Think” items that were highly practiced were recalled at a lower rate than Baseline words. This below-baseline memory result for No-Think items provided evidence that forgetting can be an active, controllable process.

A follow-up fMRI study using the same task revealed further evidence for an active form of suppression (Anderson et al., 2004). It was found that bilateral prefrontal brain regions were more active during No-Think compared to Think trials. This frontal control network increase in activation was also associated with a decrease in hippocampal blood-oxygen-level dependent (BOLD) signal, suggesting that prefrontal cortex plays an active role in inhibiting memory formation in medial temporal regions. Another study that used non-verbal memory found similar effects, although fMRI activity was lateralized to the right hemisphere in this case (Depue et al., 2006), though it is unclear whether it is the nature of the stimuli or key elements of successful memory suppression that produce this lateralization. Regardless, these findings support an active suppression mechanism for the control of forgetting, athough some studies have proposed mechanisms for non-inhibitory explanations of the TNT effect such as interference (e.g., Tomlinson et al., 2009; Benoit and Anderson, 2012).

Many subsequent studies have replicated the initial behavioral findings of the TNT paradigm (Bergström et al., 2007, 2009a; Depue et al., 2007, 2013; Anderson and Levy, 2009; Joormann et al., 2009; Paz-Alonso et al., 2009; Lambert et al., 2010; Anderson et al., 2011), although some have failed to find below-baseline forgetting in the No-Think condition (Bulevich et al., 2006; Bergström et al., 2009b; Mecklinger et al., 2009).

Electrophysiological studies employing the TNT paradigm have found early frontal event-related potential (ERP) components that may reflect the increased activation seen with fMRI. Though the ERP results have been varied, they have consistently found a reduction in the late left parietal positivity, also known as the memory retrieval effect, an ERP component associated with successful memory retrieval (Allan and Rugg, 1997), for No-Think compared to Think trials (Bergström et al., 2007; Mecklinger et al., 2009; Depue et al., 2013). However, in most TNT experimental designs, the instruction cue (i.e., “Think” or “No-Think”) and the cue word are presented simultaneously, making it difficult to disentangle instruction-based responses from the actual memory-related inhibition of the particular item. A few studies have temporally separated the instruction cue from the memory item, and have found that this manipulation behaviorally increases successful forgetting, and also reveal distinct and separable cue and item ERP effects of voluntary suppression (Hanslmayr et al., 2009, 2010). We adopted a similar design to enhance memory suppression and examine auditory memory item responses apart from instruction cue responses.

Recent investigations have begun to examine the neural oscillations involved in the control of memory (Depue et al., 2013; Anderson and Hanslmayr, 2014; Ketz et al., 2014), with a focus primarily on theta band (4–8 Hz) activity. Increased theta power has been commonly associated with successful memory retrieval (Burgess and Gruzelier, 1997), but to what extent theta oscillations are important for the control of memory processes (i.e., memory retrieval and suppression) remains unclear. Results from the available studies have yielded seemingly conflicting results on this topic. In a picture-face pairing modification of the TNT task, Depue et al. (2013) found increased theta for No-Think items in centro-parietal regions, while in a separate study, researchers from the same group (Ketz et al., 2014) found increased theta power for Think items. Another study by Waldhauser et al. (2015) reanalyzed the data from Hanslmayr et al. (2009) ERP findings to examine the oscillatory effects during intentional memory retrieval and inhibition. Using source localization, they found a decrease in theta power in medial temporal lobe for No-Think compared to Think during active memory inhibition. While a few key differences in the experimental design and analysis methods of these studies may account for the varied findings, the question of just how theta is involved in these memory processes remains unanswered.

To date, all of the TNT studies have utilized a variety of visual stimuli, including faces and scenes (Depue et al., 2006, 2007), and neutral and emotional words (van Schie et al., 2013). Memories that are rooted in other sensory domains have not yet been examined. In the present study, we examine intentional memory suppression with a modified version of the TNT task using electroencephalographic (EEG) to define the behavioral and neural correlates of the cognitive control of auditory memory. We hypothesized that the mechanisms of memory inhibition in the TNT paradigm are modality independent. Specifically, we predicted that the reduction of the memory retrieval effect, which is the most consistent and robust finding in the TNT ERP literature and is characterized by a left-lateralized parietal positivity occurring between 400 and 800 ms in the visual domain (Rugg, 1995), will be reduced for No-Think words as seen in previous TNT studies (Bergström et al., 2007; Mecklinger et al., 2009; Depue et al., 2013), but will have a longer onset latency because auditory word stimuli unfold over time.

## Materials and Methods

### Participants

A total of 18 English-speaking undergraduate students (10 F, 18–25 years) participated for course credit or monetary compensation ($12/h). All participants reported an absence of neurological and psychiatric disorders, normal or corrected-to-normal vision, and provided written informed consent approved by the University of California, Berkeley Committee for Protection of Human Subjects.

### Stimuli and Design

**Figure 1:**
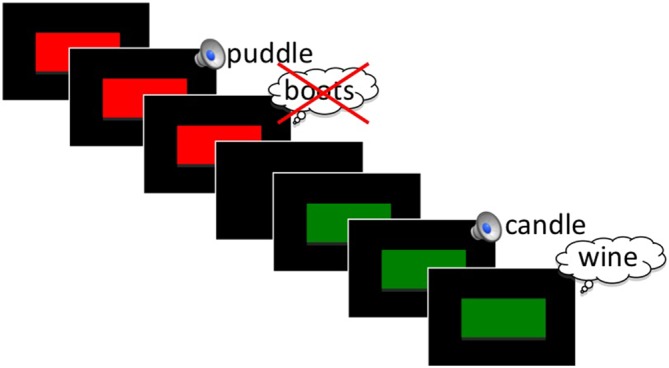
**Schematic of the Think/No-Think (TNT) phase of the experiment for an example No-Think (red) and Think (green) trial.** See text for stimulus timing.

We developed a modified version of Anderson et al. (2004) visual TNT paradigm. In this version all word stimuli were presented in the auditory domain. Only the instruction cues were presented visually (see Figure 1).

Auditory stimuli consisted of 48 pairs of English nouns developed for this experiment, paralleling the procedures used in previous TNT studies. All word pairs were chosen to have a weak semantic relationship. The second word of each pair fit into a semantic category, such that it could be probed with a category and stem-completion task for the independent probe subsequent memory test. Unlike previous studies, we chose to use non-exemplar words. That is, instead of using the most common example of a category (e.g., “water” for category “beverage”), we used a less common item from that category (e.g., “wine”). Of the 48 word pairs, six were designated for practice, and the remaining 42 were split evenly into 3 groups of 14 for Think, No-Think, and Baseline conditions. Eighteen repetitions of each Think and No-Think cue word were used in the TNT phase of the experiment.

Auditory word stimuli were recorded using a Zoom H2 microphone and edited using Adobe Audition 3.0, where words were cut and their volume normalized.

### Procedure

#### Learning

In the initial learning phase of the experiment, all 48 word pairs were presented with 1000 ms between the onset of the first and second word of each pair and a 6600 ms inter-trial interval. Participants were asked to try to learn the pairs such that if they were given the first word, or “cue word”, they would be able to respond with the second, or “target word”.

#### Recall

Recall memory was tested immediately following the initial study phase. Subjects were given the cue word of each pair and asked to respond with the target word, receiving feedback on each trial. They were asked to continue studying unlearned word pairs until each item was recalled correctly exactly once, for a learning criterion set at 100%. Recall for this phase was self-paced.

#### TNT

In a pilot study that consisted of fewer word repetitions (five repetitions, 20 word pairs per condition), we failed to find any behavioral indication of memory suppression, although we did observe similar ERP effects to what is reported here. We increased repetition number in the present study in an attempt to maximize the chances of producing a below-baseline behavioral suppression effect. With the exception of practice items, all Think and No-Think cue words were presented 18 times in a randomized fashion during this phase of the experiment. All pairs had been previously learned in the intermediate recall phase of the study, based on a 100% learning criterion. Cue words designated as Baseline items were not presented, but rather retained for use in the later subsequent memory tests to serve as a measure of passive forgetting. On each TNT trial, participants viewed a visual instruction cue that appeared centered on the screen preceding the auditory presentation of the cue word. We separated the cue and word presentation because anticipation of a to-be-inhibited item has been shown to have differential effects on ERPs (Hanslmayr et al., 2009) and also to increase later forgetting of items (Hanslmayr et al., 2010). Participants were instructed to either silently recall (green box; Think words) or inhibit (red box; No-Think words) the target word after hearing the cue word. It was stressed to all participants that on No-Think trials, it was important to keep the target word out of mind and not to think about it even after the trial was over. All participants were instructed to directly suppress, and not merely to substitute the to-be-suppressed word with an alternate item. This instruction was given because intentional forgetting can be achieved with either thought substitution or by direct suppression of items (Hertel and Calcaterra, 2005; Bergström et al., 2009a), which may produce differing brain mechanisms (Benoit and Anderson, 2012). In each trial, the instruction cue appeared for 900 ms with a 100 ms jitter before the first word of each Think or No-Think pair was presented (Figure 1). The instruction cue remained on the screen for the entirety of the word presentation. Inter-trial interval was 3600 ms with a 500 ms uniformly distributed jitter.

#### Subsequent Memory

Subsequent memory was tested using both the independent and the same probe method developed from Anderson and Green (2001) TNT visual paradigm. In the independent-probe test, subjects were given a semantic category followed by a letter and were instructed to respond with a word that fit into that category and began with that letter. Participants were given 5000 ms to respond before the automatic advancement to the next trial. Each semantic category and letter primed for a particular target word from the initially learned list. In the same probe test, subjects were auditorily presented with the first word of each pair and asked to respond with the second word of the pair, regardless of previous instruction in the TNT phase of the experiment. Participants were given 5000 ms to respond before the automatic advancement to the next trial. Subject responses were recorded using a Zoom H2 microphone. In post-processing, auditory response onsets and offsets were manually marked using a combination of the raw audio trace and time-frequency representation (for similar methods, see Flinker et al., 2011; Piai et al., 2013).

### Electrophysiological Recordings and Analysis

Scalp EEG was recorded at 1024 Hz from a 64-channel active electrode system (Biosemi; 10–20 system positions). Additional electrodes were used for reference (earlobes), and to record ocular (EOG) activity.

Offline, the data were preprocessed and analyzed using MATLAB 2011b, custom scripts, and the EEGLAB toolbox (Delorme and Makeig, 2004). Independent-component analysis (ICA) was used to remove vertical and horizontal EOG activity. Excessively noisy electrode channels were determined by visual inspection and replaced using spherical spline interpolation of the voltage from surrounding electrodes. The data were then re-referenced using current source density (CSD). We computed the CSD reference using the MATLAB implementation of a spherical spline algorithm (Perrin et al., 1989; Kayser and Tenke, 2006) to obtain the second spatial derivative of the scalp voltage (μV/m^2^ units; flexibility parameter m = 4; smoothing parameter *λ* = 5 × 10^-5^). Positive values of the CSD indicate local current flow out of the skull and negative values indicate current flow into the skull. The CSD transformation allows for a greater degree of independence from the location of reference electrode(s) (Tenke and Kayser, 2012; Luck, 2014) and provides a more focal spatial estimation of the underlying cortical activity (Gevins, 1989; Nunez and Pilgreen, 1991). After computing the CSD on all electrodes, we removed those at the edges from further analysis since CSD reference estimations rely on surrounding electrodes. This left 41 channels for analysis. The data were then bandpass filtered between 0.1 and 35 Hz and downsampled to 120 Hz.

Epochs were created for word-locked activity for Think and No-Think conditions. All trials containing activity greater than 100 uV were removed and the remaining trials were subjected to an iterative artifact rejection process that removed any trials containing data that exceeded five standard deviations from the mean of all data at each time point. This was done iteratively until no trials remained that fit that criterion. The resulting mean number of artifact-free trials were 187 (min: 125, max: 233) and 188 (min: 127, max: 231), for Think and No-Think respectively.

Think and No-Think ERPs were created from the artifact-rejected data. Word-locked trials were baseline corrected from −100 to 0 ms prior to the visual cue stimulus onset and then averaged within each subject. We chose to use a pre-cue baseline because otherwise the word-locked activity might be confounded with post-cue activity.

Cue-locked activity preceding the auditory cue word onset was subjected to the same methods described above. The focus of this manuscript is on auditory memory suppression and retrieval so only word-locked activity is presented in the main text. For the details of cue related effects, please see “Supplementary Material”.

Time-frequency analysis of theta power during cue word presentation and memory retrieval/inhibition was calculated using artifact free epochs, and the timecourse of theta power was estimated by applying the Hilbert transform to bandpass filtered data (4–7 Hz). The timecourses were normalized by computing the relative change vs. a pre-cue interval of −300 to 0 ms. This baseline was chosen to be longer than one full cycle of the slowest theta frequency (4 Hz), yet as short as possible as to minimize the impact of the previous trial. Previous studies have used a shorter baseline for analysis of evoked theta (Bastiaansen et al., 2002; Burgess and Ali, 2002; Kamarajan et al., 2008; Mu et al., 2008). We focused on the Theta band based on its known importance in memory processing.

### Statistical Analysis

A multi-step permutation method was used to quantify differences in ERPs between the Think and No-Think conditions during the TNT phase of the experiment. The null hypothesis tested against is that there is no difference in scalp-evoked activity due to condition.

For each subject, we first computed 2000 null ERPs for each condition at each of the 41 channels. These null ERPs were obtained from subsets of trials independently drawn from the larger set of the combined trials from both conditions. The number of trials drawn from the combined set for each condition was the same as the number of trials in that condition. Each of the 2000 subsets of trials in each condition was baseline corrected using the same pre-cue baseline period described for the true ERPs and averaged to obtain a null ERP.

Following this, a set of 50,000 null difference waves (No-Think—Think) was computed for each subject at each channel. These difference waves were obtained by subtracting one randomly selected null Think ERP from another randomly selected null No Think ERP. True difference waves were also obtained for each subject using unshuffled trials.

The true and null data were then submitted to a two-tailed test based on the cluster mass statistic (Bullmore et al., 1999). All time points between 0 and 1500 ms following word stimuli at the 41 scalp electrodes were included in the test (7380 total comparisons). First, 50,000 across subject averages were computed using the 50,000 null difference waves in each subject. *T*-scores were then computed for each null grand average difference wave by comparing it to the entire distribution of null grand average difference waves at every time point. This results in 50,000 sets of 7380 (41 channels × 180 time points) *t*-scores.

For each set, all *t*-scores corresponding to uncorrected *p*-values of 0.01 or less were formed into clusters with any neighboring such *t*-scores. Electrodes within approximately 5 cm of one another were considered spatial neighbors and adjacent time points were considered temporal neighbors. The sum of the *t*-scores in each cluster is the “mass” of that cluster and the most extreme cluster mass in each of the 50,000 sets of tests was recorded and used to estimate the distribution of the null hypothesis.

Clusters were then obtained from the true data and the percentage of null cluster masses greater than each true data cluster mass was taken as the corrected *p*-value for that cluster. The *p*-value of the cluster was assigned to each member (time-channel point) of the cluster and points that were not included in a cluster (due to small *t*-score) were not given a *p*-value. Differences between conditions with a corrected *p*-value less than 0.05 were considered significant.

Statistics for theta power time-courses were calculated using the same method as described above for ERPs, applied to pre-cue baseline word-locked average theta power.

This permutation test was used instead of mean amplitude analysis of variance (ANOVAs) because it provides much better spatial and temporal resolution than conventional ANOVAs while at the same time maintaining weak control of the family-wise alpha level at 0.05. The cluster mass statistic was chosen for this permutation test because it has been shown to have relatively good power for ERP effects (Groppe et al., 2011). See Luck (2014) as well as Maris and Oostenveld (2007) for further review of this method.

Because this method provides exact and distinct periods of significance across all channels, we approximate the range of temporal significance in the text of this manuscript. Exact values of significance can be seen in the main text figures, as well as a more detailed view in the Supplementry figures.

## Results

### Behavior

#### Accuracy

**Figure 2:**
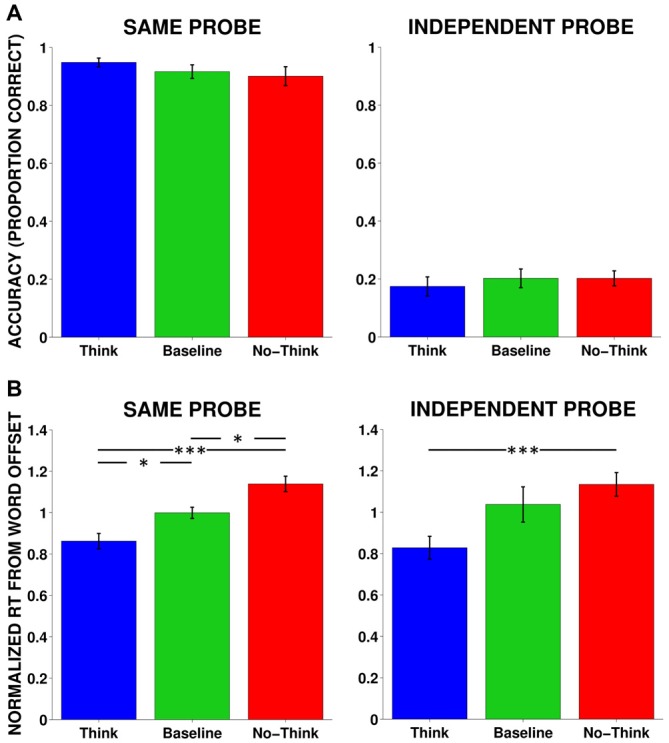
**Accuracy and response time (RTs) averaged across all subjects. (A)** Behavioral accuracy (proportion correct) in the same and independent probe tests. No significant results were found. Same Probe: *F*_(2,34)_ = 1.87, *p* = 0.17, *η*^2^ = 0.14, Independent Probe: *F*_(2,34)_ = 0.39, *p* = 0.68, *η*^2^ = 0.02. **(B)** RT for normalized mean responses from word offset to the onset of the correct response for same probe and independent probe tests. Same Probe: *F*_(2,34)_ = 10.92, *p* < 0.001, *η*^2^ = 0.46, Independent Probe: *F*_(2,34)_ = 3.78, *p* < 0.05, *η*^2^ = 0.24. *p < 0.05, **p < 0.01, ***p < 0.001.

We conducted one-way repeated-measures analysis of variance (rmANOVAs) on accuracy for same probe and independent probe tests. Accuracy was defined as the proportion of items that received the appropriate target word response. In the same probe test, although numeric values of the memory conditions were in the predicted direction (i.e., highest memory for Think, followed by Baseline and No-Think, respectively), no significant differences were found for either the same probe (*F*_(2,34)_ = 1.87, *p* = 0.17, *η*^2^ = 0.14) or independent probe (*F*_(2,34)_ = 0.39, *p* = 0.68, *η*^2^ = 0.02) tests (Figure 2A).

#### Response Time

We examined the strength of memory facilitation and suppression using a RT metric. We analyzed RTs for correct answers elicited from the same and independent probe tests. Since auditory cue words varied in length, we measured RTs from the offset of the cue word to the onset of the correct response. We normalized responses with each individual subject by dividing each RT by the average RT across all three conditions. For the same probe test, using one-way rmANOVAs, we found a significant effect of memory condition when measuring normalized mean latency from cue-word offset (*F*_(2,34)_ = 10.92, *p* < 0.001, *η*^2^ = 0.46), such that responses were fastest for Think items, followed by Baseline and No-Think items, respectively. All memory conditions were different from each other and in the predicted direction, supporting both facilitatory and inhibitory effects (Figure 2B). Although we had lower overall accuracy for the independent probe, we found a similar relationship among the RTs: *F*_(2,24)_ = 3.78, *p* < 0.05, *η*^2^ = 0.24, where correctly remembered Think items were more quickly recalled than correctly remembered No-Think items. Although the suppression effect between Baseline and No-Think RTs did not reach significance for the independent probe, a significant linear contrast in the predicted direction suggests that memory control was systematically manipulated according to instruction, *F*_(1,12)_ = 18.11, *p* < 0.001, *η*^2^ = 0.60.

### ERPs

**Figure 3:**
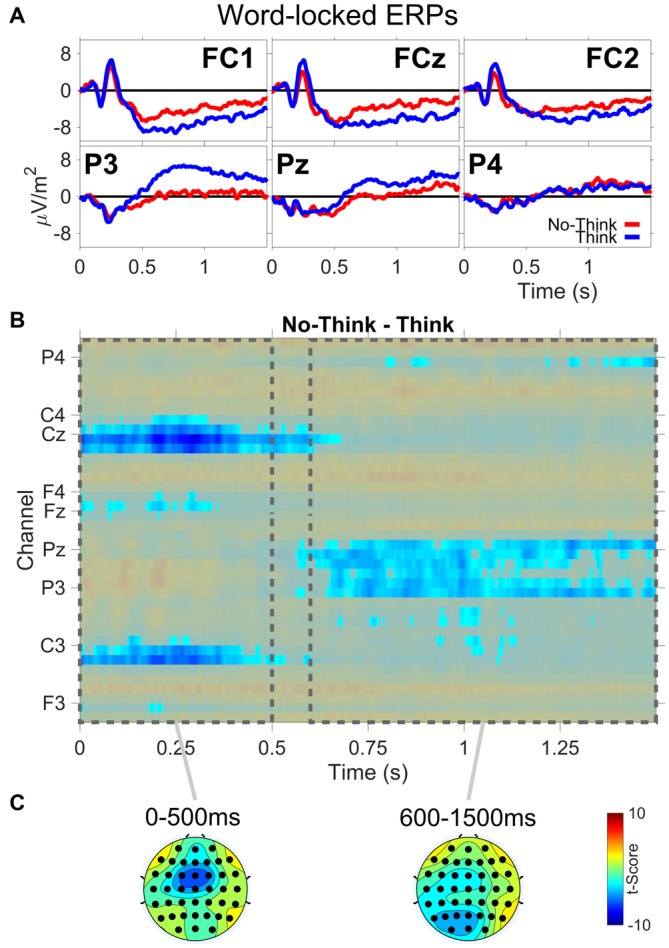
**Word-locked event-related potential (ERP) data. (A)** ERP waveforms from three frontal (FC1,FCz,FC2) and three parietal (P3,Pz,P4) electrode channels. **(B)** No-Think—Think significant *t*-scores (*p* < 0.05) at all electrodes and time points. **(C)** Topographies of *t*-scores for No-Think—Think, averaged over the duration of two time periods (0–500 ms and 600–1500 ms).

The ERP results are from the TNT phase of the experiment. Figure 3 shows: (A) ERP waveforms for three frontal (FC1,FCz,FC2) and three parietal electrodes (P3,Pz,P4); (B) results from the permutation and cluster analysis; and (C) topographies of *t*-scores for No-Think—Think, averaged over the duration of two time periods (0–500 ms, 600–1500 ms). Permutation and cluster analysis highlighted two main effects. First, from word onset until about 500 ms, we found a centralized Think > No-Think effect during the auditory word presentation. Second, we observed a sustained increase in Think compared to No-Think trials starting around 600 ms at left lateralized posterior electrode sites which was sustained through the end of the trial, reliably demonstrating the parietal memory retrieval effect observed in previous TNT studies (e.g., Bergström et al., 2007; Mecklinger et al., 2009). Critically, the memory retrieval component is almost completely abolished for No-Think trials, indicating successful memory suppression.

**Figure 4:**
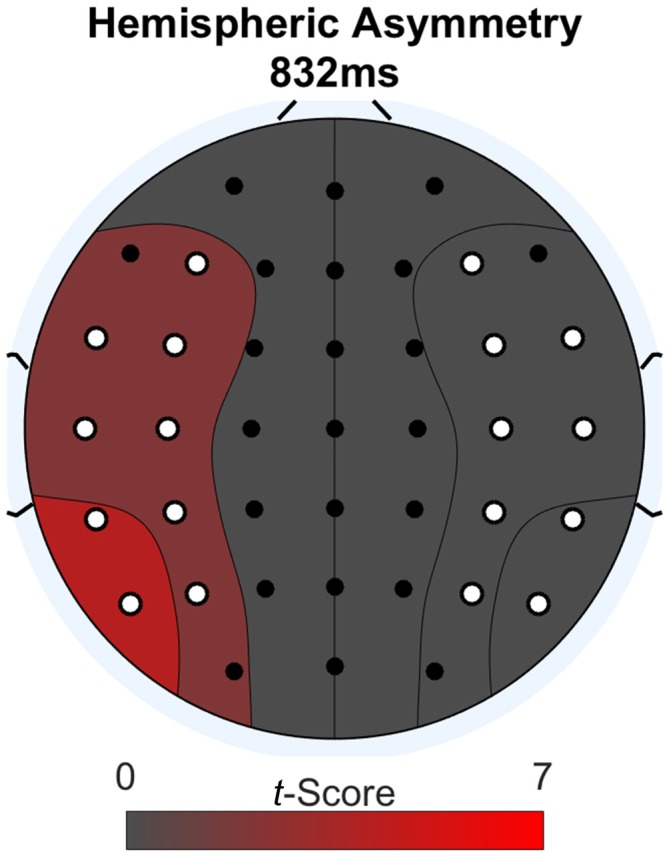
**Lateralization effect during word-locked ERP activity at the maximal condition difference.** Difference scores larger than the opposite side of the head are depicted in red. Electrodes with significant differences *p* < 0.05 are colored in white.

We further investigated the word-locked ERPs to determine if the late parietal positivity was lateralized. We did a second permutation analysis to address this issue. The methodology was the same as for the previous analysis, except the two conditions compared were: (1) difference waves between Think and No-Think words; and (2) these same difference waves mirrored across the midline electrodes. Figure 4 shows a topographic plot of any hemispheric asymmetries at the time point of maximal difference between conditions (832 ms after word onset). We determined this time point by summing the absolute value of the *t*-scores across channels for every time point during the word epoch and selecting the time point with the greatest value after smoothing using a 100 ms zero-phase moving average. Areas on the plot colored red have a numerically greater condition difference in that hemisphere compared to the other. All electrodes with a significant laterality difference are marked in white. We found a significant lateralization effect, such that condition differences (Think > No-Think in the observed data), were larger over the left region compared to right (*p* < 0.05) at the point of maximal condition difference.

### Theta Power Timecourse

**Figure 5:**
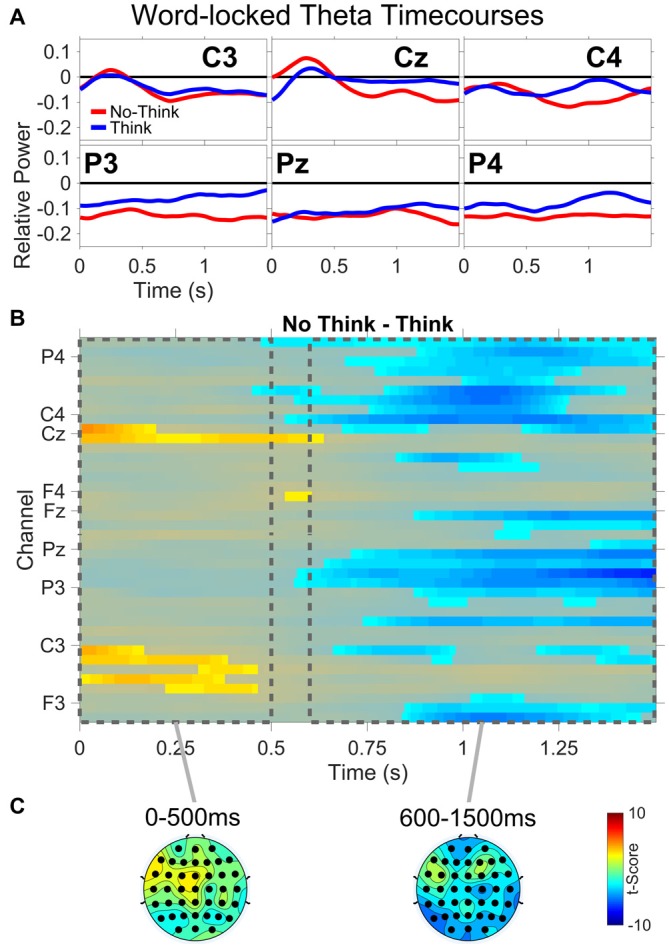
**Word-locked theta power. (A)** Average power time courses for three central (C3,Cz,C4) and three parietal (P3,Pz,P4) electrode channels. **(B)** No-Think—Think significant *t*-scores (*p* < 0.05) at all electrodes and time points. **(C)** Topographies of *t*-scores for No-Think—Think, averaged over the duration of two time periods (0–500 ms and 600–1500 ms).

All theta results are from the TNT phase of the experiment. Figure 5 shows: (A) word-locked average theta power time courses for three central (C3,Cz,C4) and three parietal electrodes (P3,Pz,P4); (B) results from the permutation and cluster analysis for theta power; and (C) topographies of *t*-scores for No-Think—Think, averaged over the duration of two time periods (0–500 ms and 600–1500 ms). Permutation and cluster analysis demonstrated two main findings. From word onset to approximately 500 ms, a No-Think > Think difference at central and left central electrode sites was sustained during auditory word presentation (see “Supplementary Material” for a detailed view of each significant channel and time point). This was immediately followed by a sustained Think > No-Think difference which remained through the end of the trial.

## Discussion

The aim of the study was to determine whether auditory TNT produces similar behavioral and electrophysiological effects to that observed in the visual domain. Our data provide evidence that the act of engaging in memory inhibition extends across modalities.

We employed a relatively large number of inhibition repetitions (18 reps × 14 No-Think words = 300 total inhibition attempts) in an effort to maximize the ability to produce the classic TNT effect. Note that, similar to Anderson and Green (2001), we did not find any reliable suppression effects in pilot data based on only five repetitions (5 reps × 20 No-Think words = 100 total inhibition attempts). Evidence from experimental manipulations as well as self-reports of previous experience with inhibition have suggested that an increase in inhibition practice and not just inhibition of a particular item improves intentional forgetting performance (Anderson and Levy, 2009; Bergström et al., 2009a). For this reason, we hypothesized that increasing the number of total inhibition trials to 300 would likely produce below-baseline forgetting for No-Think items. We further attempted to maximize the likelihood of a classic TNT behavioral effect by temporally separating the instruction cue and memory cue (Hanslmayr et al., 2010).

However, in our first behavioral analysis we failed to find significant differences in accuracy for both same and independent probe tests using auditory stimuli. The lack of a significant suppression effect in the same probe test may be a result of near-ceiling performance exhibited by the young adults that participated in the experiment, but this cannot explain the null effect in the independent probe test. The low accuracy rates in the independent probe test, which hovered around 20% and are much lower than in other studies, typically above 80% (e.g., Anderson and Green, 2001; Anderson et al., 2004; Hanslmayr et al., 2009), and may be due to our decision to use non-exemplars as target words. We used words that were less common examples of the semantic categories they belonged to. As a result, the use of target words in this categorical cue with stem completion test was very low across conditions and may have precluded observing an accuracy finding due to a floor effect.

Though we did not find differences in the accuracy data, RTs have been shown to be a reliable measure of memory strength in the TNT paradigm (Waldhauser et al., 2012). Examination of the RTs made in both subsequent memory tests reliably provided evidence for direct facilitation and inhibition of auditory memory strength. Importantly, in the same-probe test, we found that RTs were faster for Think and slower for No-Think items compared to the Baseline measure, confirming auditory memory manipulability using the classic visual TNT paradigm structure. These authors note that this lack of significant below-baseline response latency for No-Think items may appear as evidence against successful suppression, but we argue that the low accuracy to the independent probe, coupled with the fact that only 13 out of 18 subjects responded with enough correct responses to be considered in the analysis, makes the independent probe test inadequate to draw conclusions about an absence of suppression. For these reasons, we cannot conclusively establish the argument for inhibition in this study, although the evidence points to that conclusion. Furthermore, the significant linear contrast in the predicted direction and trend toward significance is supportive that memory is being manipulated in a systematic way according to task instruction. Because we were not able to produce complete forgetting, but instead a subtler index of memory suppression, these behavioral findings suggest that auditory memory may be more difficult to manipulate than visual memory, but based on the electrophysiological findings the same neural mechanisms are engaged.

Our behavioral results imply that it may take more sensitive measures to examine memory inhibition in audition. Moderate activation of memories has been shown to render those memories more easily inhibited, as compared with weaker or stronger memories (Detre et al., 2013). This may be the cause of our inability to produce changes in accuracy in the No-Think condition in this study. It may be that auditory memory is more resistant to suppression, or that the material or design used in this study in particular is not ideal for examining memory manipulation.

Turning to the ERP analysis, word-locked activity produced electrophysiological effects similar to that observed in the visual domain, specifically the memory retrieval effect (Bergström et al., 2007; Mecklinger et al., 2009; Depue et al., 2013). There was an initial Think > No-Think centralized positivity difference that began at word onset and remained through the auditory word presentation. This was followed by a localized left-lateralized parietal memory retrieval effect (Allan and Rugg, 1997). This effect, consisting of an amplitude increase for Think words has been proposed to represent the successful memory retrieval of the pair word. This index of successful memory retrieval was significantly reduced in the No-Think trials, indicating successful memory inhibition.

In addition to supplying evidence that auditory memory can be manipulated in much the same way as visual memory, we offer evidence relevant to the debate of whether theta oscillatory power in the TNT paradigm is a marker of successful memory (Ketz et al., 2014), or rather reflects higher-level cognitive control of multiple memory processes (Depue et al., 2013). Our data suggest that both processes may be engaged. The early theta increase for No-Think words during the word presentation over frontal and left-lateralized frontal electrodes suggests an early control mechanism to specifically target No-Think words. This may be the ERP correlate of the No-Think > Think BOLD response seen in fMRI TNT paradigms in the visual domain (Anderson et al., 2004; Depue et al., 2006). Based on the results of this study, one interpretation may be that frontal control mechanisms have to be more strongly engaged for No-Think trials during the auditory word presentation. However, once the cue word stops being actively presented, theta power becomes stronger for Think compared to No-Think in a widespread difference emerging around 600 ms, which may reflect the successful memory retrieval of the Think words.

This present study provides encouraging evidence for the ability to inhibit auditory memories using the TNT paradigm. The EEG effects shown here are in line with those presented in the visual domain; visual studies report a left-lateralized Think positivity over parietal areas, and No-Think increases over frontal electrode sites, both of which we report here. However, we caution that although the electrophysiological data strongly mirrors that of visual TNT results, the fact that we did not find below-baseline forgetting in accuracy measures for either subsequent memory test, could reflect a lack of true inhibition in our task. Why our manipulation did not produce the expected result of forgetting of No-Think words below baseline (as measured by accuracy) is unclear. The authors believe the most plausible explanation is that auditory memory may be more difficult to manipulate than that of the visual domain, and this may have contributed to both the ceiling effects of the accuracy measurements seen in the same test, as well as the inability find significant classic TNT accuracy effects. With this in mind, the combination of RT and ERP effects still do provide evidence of active inhibition in our auditory TNT paradigm, suggesting that the auditory mechanisms are similar to that observed in the visual domain.

One of the most attractive aspects of the TNT model is that items that are to-be-forgotten are ideally forgotten below baseline levels of forgetting. We see potential benefits to this method in terms of adapting it for clinical purposes. However, to date, all TNT experiments have used visual stimuli, leaving a missing piece in our understanding of memory inhibition. For instance, traumatic memory recall in PTSD patients is likely not limited to the visual domain, and may include auditory, olfactory, tactile, and even taste information. This study extends the current literature by suggesting that all modalities of memory may be able to be suppressed, perhaps to different degrees. Further research combining or directly contrasting different memory modalities may provide insight into how real-life memories might be successfully forgotten.

## Author Contributions

MEC carried out collection and analysis of the data, and drafted the initial version of the manuscript. RTK contributed significantly to data interpretation and provided critical revisions. Both authors have approved the final version of the manuscript and agree to be accountable for all aspects of the work.

## Funding

This work was supported by the NINDS Grant R37NS21135 (RTK), the Nielsen Corporation (RTK), and the NSF Graduate Research Fellowship (MEC).

## Conflict of Interest Statement

The authors declare that the research was conducted in the absence of any commercial or financial relationships that could be construed as a potential conflict of interest.
