# Supplementary material for: Behavioral and EEG Evidence for Auditory Memory Suppression
Source: Front Hum Neurosci. 2016 Mar 30;10:133. doi: 10.3389/fnhum.2016.00133 (PMC4811890; doi:10.3389/fnhum.2016.00133)
Supplement: Supplementary file 1 [file DataSheet_1.docx]

***Supplementary Material***

**Behavioral and EEG evidence for auditory memory suppression**

**Maya E. Cano**^1^*** & Robert T. Knight**^1,2^

^1^Helen Wills Neuroscience Institute, University of California, Berkeley, CA, USA

^2^Department of Psychology, University of California, Berkeley, CA, USA

*** Correspondence:**

Maya E. Cano, PhD

Knight Lab

Helen Wills Neuroscience Institute

University of California

132 Barker Hall

Berkeley, CA 94720-3190, USA

email: maya.cano@gmail.com

**1. Supplementary analysis**

Supplementary analyses were performed for the visual instruction cue period during the Think/No-Think phase of the experiment that preceded auditory word onset. These analyses are not included in the main text of this manuscript because they do not contribute to the focus of the manuscript, which is suppression of auditory memory and not on contributing preparatory visual instruction. The results from this period of the experiment are included for completeness. All data analysis and statistical methods performed on the cue-locked data are identical to that of the word-locked activity with the only exception being the epoch length, so that the epoch only encompassed cue activity in the absence of the auditory word. As a result, permutation and cluster analyses was performed on time points between 0 and 750ms following visual cue onset, for a total of 3690 comparisons, which resulted in 3690 (41 channels x 90 time points) t-scores for cue stimuli. *Supplementary Figures 1* and *2* show the results of these analyses.

*Supplementary Figures 3* through *5* expand on results and discussion from the main text.

**2. Supplementary figures**

**2.1. Cue-locked event-related potentials**

*Supplementary Figure 1* and *2* show the event-related potential (ERP) results from the visual instruction cue analysis. We observed a P2 component difference, with Think > No-Think at central sites. The P2 has been shown to be modulated on the basis of attentional selection of visual stimulus features (e.g., Smid, et al., 1999). Thus, the Think cue may produce this P2 enhancement effect because it is a more relevant indicator of the upcoming need to remember. We also observed sustained Think and No-Think differences during the 300-500ms time frame. Lastly, a sustained lateral frontal effect appeared by approximately 500ms, and remained through the end of the cue period. This prolonged effect may reflect relevant preparatory activity similar to what has been previously reported by Hanslmayr et al. (2009). Though some of these cue effects are similar to what has been shown previously in vision, we cannot directly compare the results to prior visual studies given our separation of cue and word trial period.

**
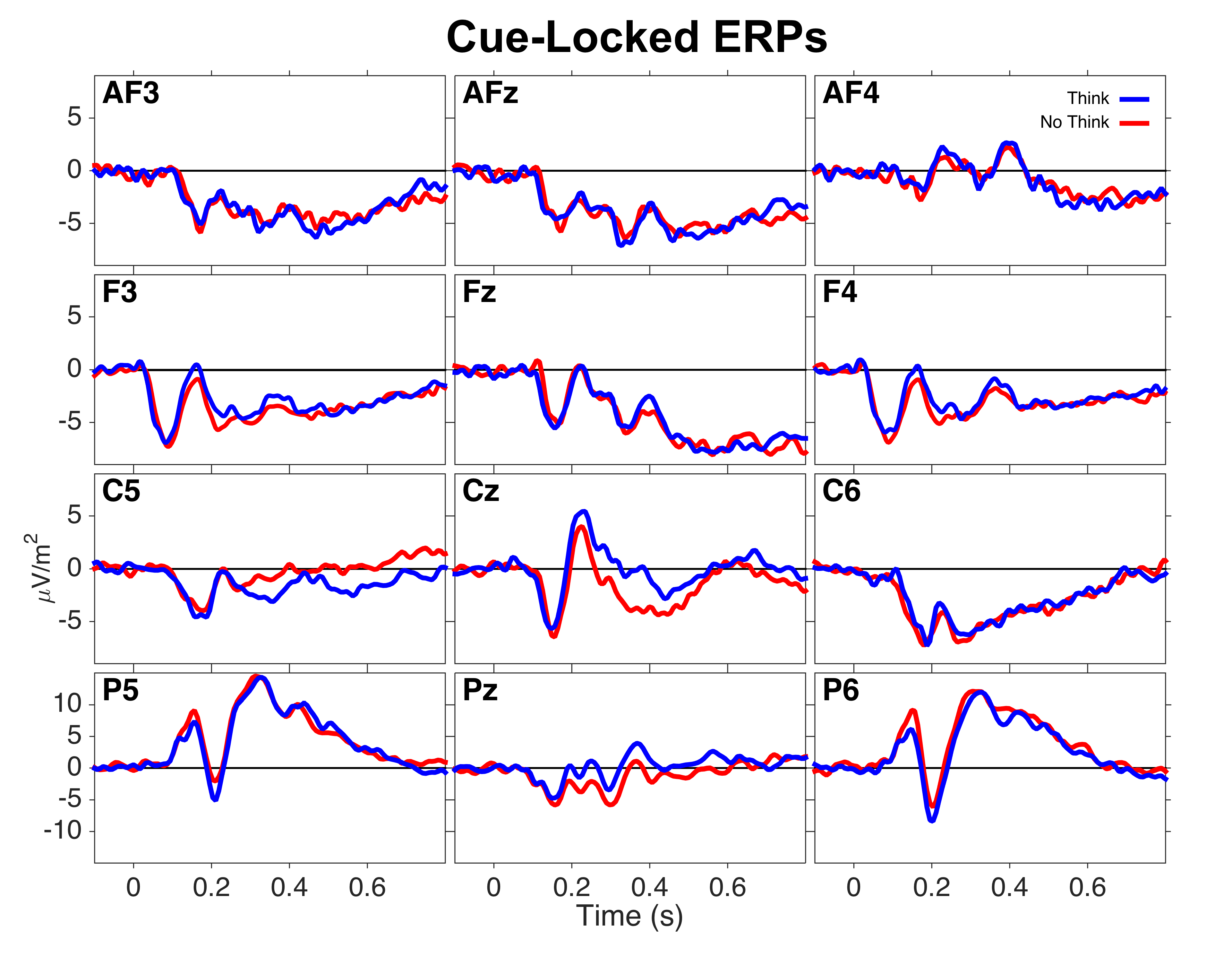
**

Supplementary Figure 1. Cue-locked ERP waveforms. ERP waveforms for three anterior (AF3, AFz, AF4), three frontal (F3, Fz, F4), three central (C5, Cz, C6), and three parietal (P5, Pz, P6) electrode channels.

**
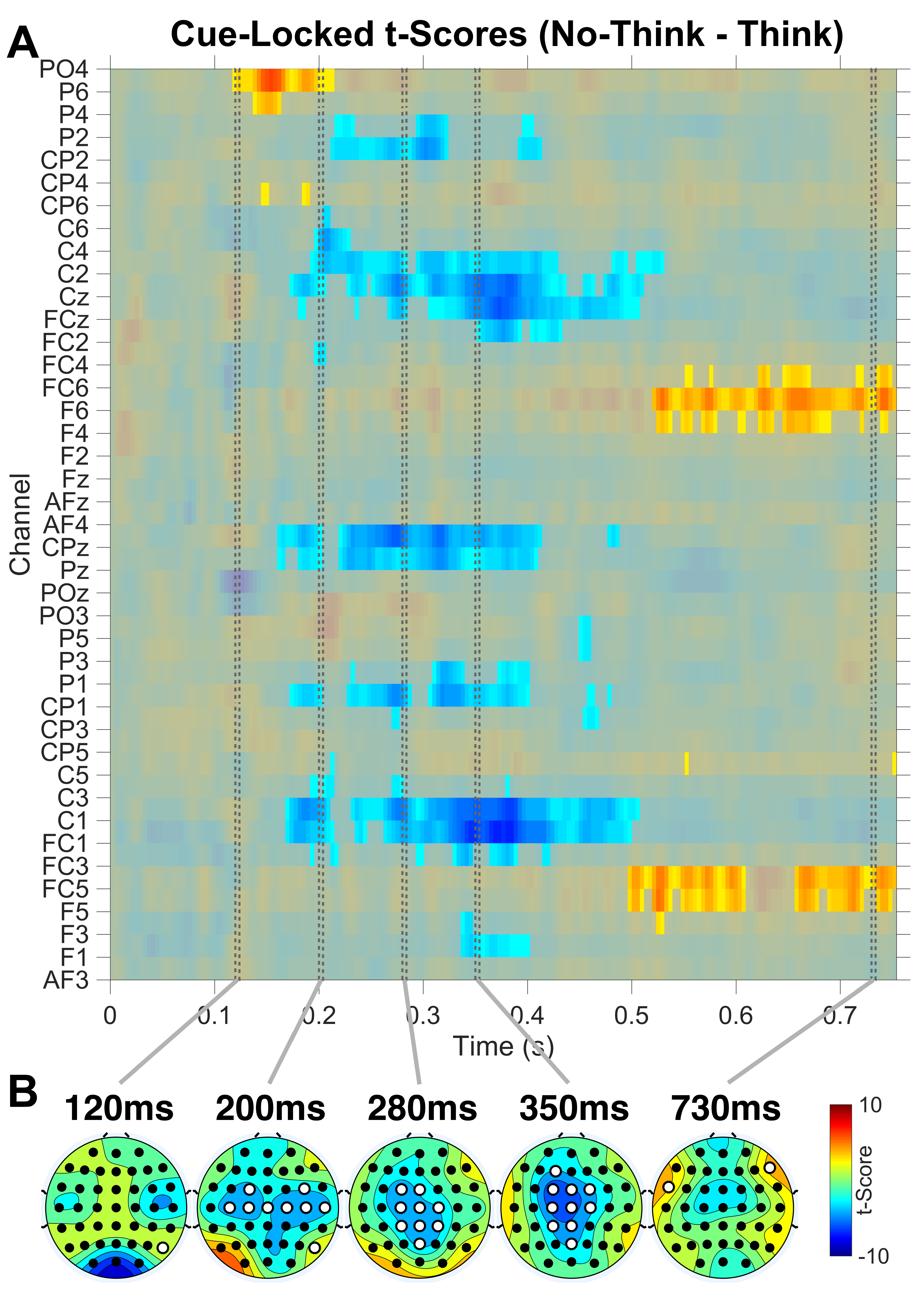
Supplementary Figure 2. Cue-locked statistical results for (A) No-Think – Think significant t-scores (*p*<0.05) at all electrodes and time points and (B) topographies at five time points showing significant condition differences.** Electrodes that show a significant difference at *p*<0.05 are marked in white.

**2.2. Word-locked permutation results**

Supplementary figures for word-locked permutation and cluster analyses for ERP and theta band results show the same data from the main text, but with all 41 electrode channels labeled for a more detailed analysis of the statistical results.

**
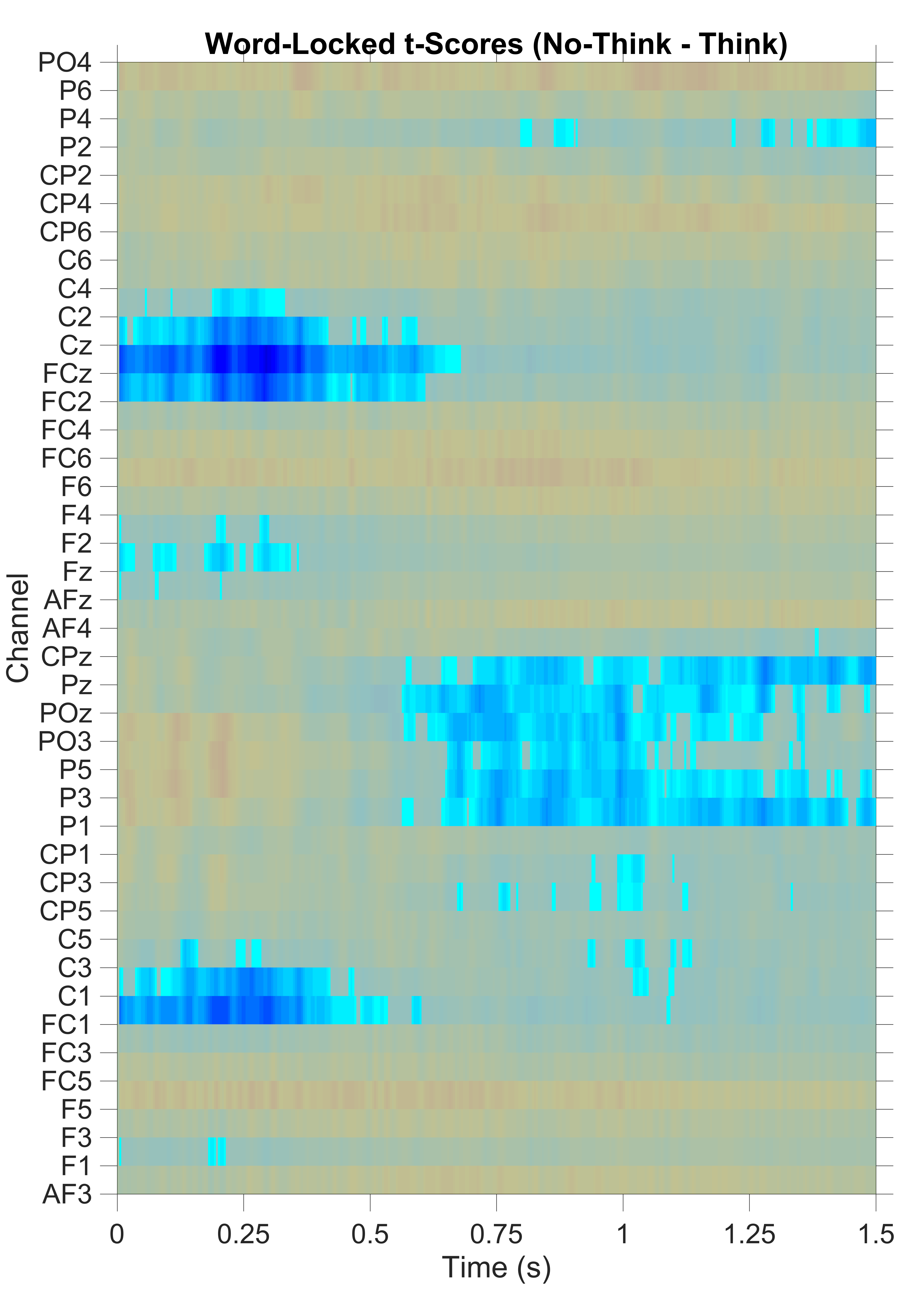
Supplementary Figure 3. ERP word-locked permutation and cluster analysis significant t-scores (*p*<0.05) for all 41 electrode channels.**

**
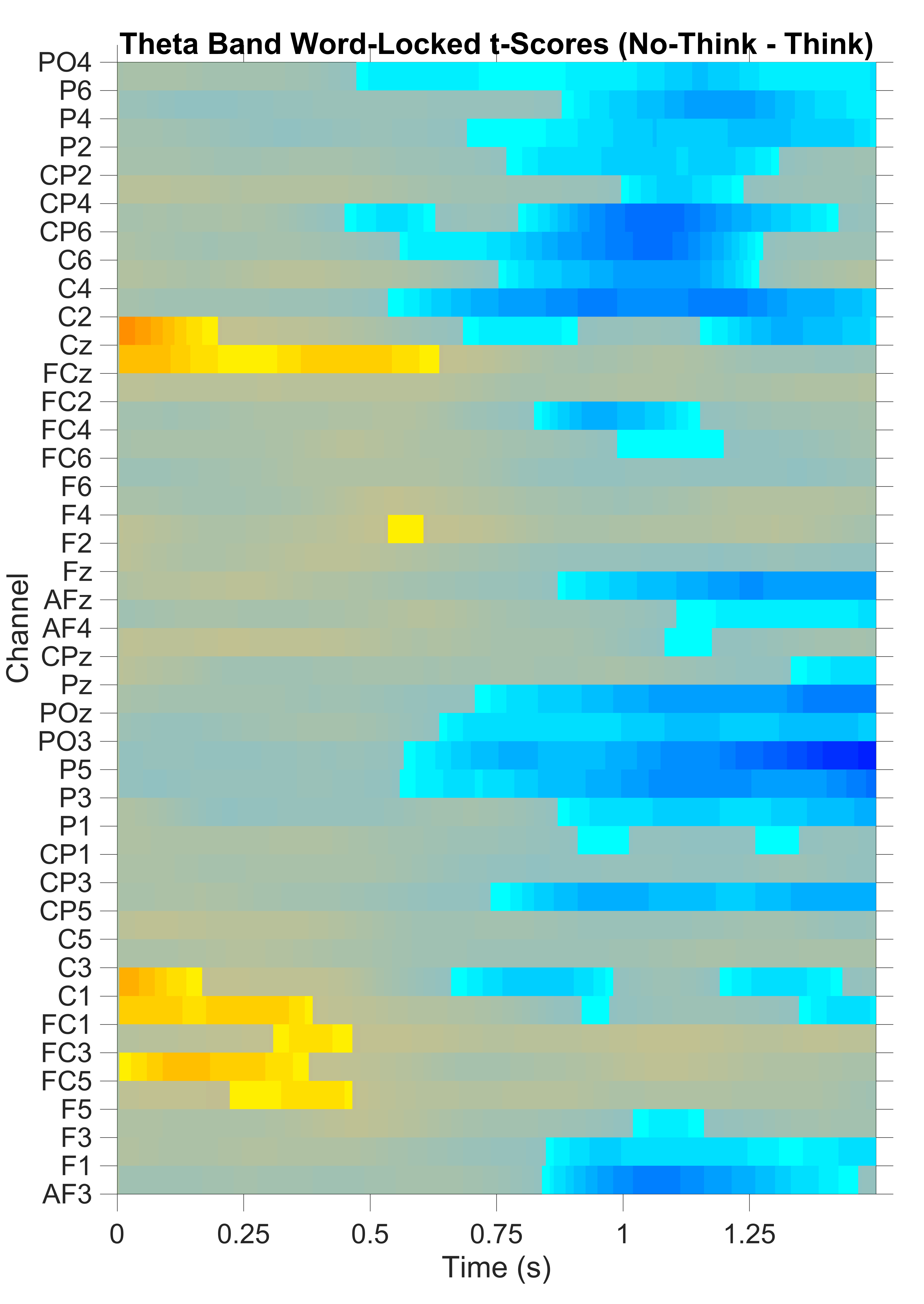
**

**Supplementary Figure 4. Theta band word-locked permutation and cluster analysis significant t-scores (*p*<0.05)** **for all 41 electrode channels.**

**3. Supplementary references**

Smid, H. G., Jakob, A., and Heinze, H. J. (1999). An event-related brain potential study of visual selective attention to conjunctions of color and shape. Psychophysiol 36, 264–279.

Hanslmayr, S., Leipold, P., Pastotter, B., and Bauml, K. H. (2009). Anticipatory Signatures of Voluntary Memory Suppression. Journal of Neuroscience 29, 2742–2747. doi:10.1523/JNEUROSCI.4703-08.2009.
